# Supplementary material for: Are carbon emissions trading and green financial instruments synergistic? -Comprehensive quantitative research based on content analysis
Source: PLoS One. 2024 Mar 7;19(3):e0298601. doi: 10.1371/journal.pone.0298601 (PMC10919601; doi:10.1371/journal.pone.0298601)
Supplement: S1 Data — (ZIP) [file pone.0298601.s001.zip › Data/地级市Z_F工作报告.zip!/╣π╢1⁄2/╓Θ║ú╩╨/└┤╫╘╣1⁄2╓┌║┼í╛┬φ┐╦╩2╛▌═°í┐_2004.docx]

各位代表：

　　现在，我代表珠海市人民政府向大会作政府工作报告，请予审议，并请各位政协委员和其他列席人员提出意见。

　　一、过去五年工作的回顾

　　１９９９年以来的五年，是珠海不断开拓创新并实现加快发展的五年。市政府高举邓小平理论伟大旗帜，践行“三个代表”重要思想，贯彻党的十五大、十六大精神，在省委、省政府和市委的领导下，在市人大、市政协的监督支持下，以“增创新优势，更上一层楼，率先基本实现社会主义现代化”统揽全局，围绕建设“三基地一中心”的发展目标，实施外向带动、科教兴市、可持续发展战略以及“经济功能区带动”、“两个大办”、“工业西进、城市西拓”等战略举措，物质文明、政治文明和精神文明建设取得了新成绩，圆满完成上级交给我市迎澳门回归的任务，全面实现市五届人大一次会议提出的工作目标，开创了珠海现代化建设的新局面。

　　国民经济持续快速增长。五年来，我们努力构筑“实业旺市”发展平台。突出抓好工业发展，各具特色的经济功能区和工业园区发展迅速，工业结构逐步调整，优势产业和优势企业进一步发展壮大，工业重型化趋势明显。农业结构调整成效显著，培育了一批农业产业化基地和龙头企业，水产养殖形成特色。旅游、商贸、房地产等服务业稳步发展，港口货物吞吐量快速增长，防范和化解金融风险工作卓有成效，金融机构存贷款规模扩大。经济发展速度逐年加快。与１９９８年对比，２００３年本地生产总值（ＧＤＰ）从２６３．５亿元增加到４７６．７亿元，年均增长１２．８％；工业总产值从４９２亿元增加到１０３６．６亿元，年均增长１９．３％；地方财政总收入从２４．３亿元增加到４９．１亿元，年均增长１５．２％。

　　科教兴市成效显著。五年来，我们全力构筑“科教兴市”发展平台。基础教育得到加强，新建４所中学高中部和初中部，在金湾区、斗门区建成７所规范化小学，薄弱学校改造基本完成，普及高中阶段教育，职业教育和成人教育加快发展，民办教育逐步规范提高。香洲区荣获省“教育强区”称号。创办大学园区，引进中山大学等一批著名高校来珠海办学或创办产学研基地，聚集了教育和科技创新资源。对珠海国家高新区布局进行调整取得显著成效，科技工业园、科技创新海岸蓬勃发展，多项考核指标进入全国高新区前列。大幅增加科技投入，支持建设国家软件产业基地、国家农业高新技术示范园、广东省集成电路设计与生产基地等，形成多层次的科技研发、示范和产业化体系。建设“广东省信息化综合试点城市”取得成效，“电子政务”全面启动，社会信息化水平明显提高。高新技术产业迅速发展，２００３年全市高新技术产品产值３４２．６亿元，是１９９８年的２．９倍，占工业总产值的３３．１％。珠海成为“全国科技进步先进市”和“广东现代化科技示范市”。

　　城市建设迈出新步伐。五年来，我们着力构筑“环境强市”发展平台。加快推进城市化进程，设立金湾区，斗门撤县改区。经修编的２００１－２０２０年城市总体规划获得国务院批准。花大力气进行基础设施建设，先后建成淇澳大桥、莲花大桥、横琴大桥、京珠高速公路珠海段、昌盛大桥、横坑大桥，完成珠海大道８座桥梁和部分农村公路危桥改造。每年改造一条城市干道，修通了一批烂尾路。建设口岸联检楼、垃圾焚烧发电厂、污水处理厂、防排洪和污水管网等市政基础设施。珠海电厂一号、二号机组投产发电。全面改造城中旧村，建成一批文明社区。改革城市管理体制，下放管理权限，推行相对集中行政处罚权试点。开展城市环境综合治理，国家级生态示范区建设成效显著。工业污染得到有效治理，空气、水体等生态环境质量保持优良，通过“全国环保模范城市”、“国家卫生城市”和“国家园林城市”复查，荣获广东省“环境保护先进市”称号。

　　改革开放成果丰硕。五年来，我们坚持不断推动体制创新和扩大开放。市场取向的体制改革向纵深推进，先后设立市产权交易中心、建设工程交易中心、商用土地交易中心、政府采购中心、人力资源开发管理服务中心、市财政国库支付中心和市政府投资建设工程管理中心，实现资源市场化配置和集中监管。改革国有企业和国有资产管理体制成效显著，建立起管人、管事、管资产相结合的三级经营管理架构，国有资产营运效益提高。加强对民营经济的政策扶持和引导服务，民营企业加快发展。改革财政管理体制，公共财政框架逐步形成。在全省率先以ＢＯＴ、ＴＯＴ方式建设和营运城市污水处理厂。全面推进金融、外贸、人事、住房和粮食流通等各项配套改革。改革平沙、红旗农场管理体制。招商引资成效显著，吸引了一批跨国公司投资珠海，五年累计利用外商直接投资３９．８亿美元。对外贸易规模扩大，２００３年进出口总额１６７．８亿美元，是１９９８年的２．８倍。珠港澳合作取得较大进展，对台招商成效明显。在国际金融市场成功回购珠海大道债券。举办第三届、第四届中国航展，对外经济、科技、文化交流得到加强。

　　人民生活水平稳步提高。五年来，我们努力推动经济社会事业全面发展，城乡居民物质和文化生活不断改善。２００３年，城镇居民年人均可支配收入１６６０２元，农渔民人均纯收入５５１７元，分别比１９９８年增长２１．８％和４５．４％。居民消费结构明显变化，住房、汽车、教育、文化、旅游等成为消费热点。公务员和离退休干部待遇逐步提高。促进就业和再就业，城镇登记失业率控制在较低水平。农村居民生产生活条件得到改善，城乡生活用电实现同网同价，村村通有线电视，斗门区农村用上达标自来水。社会保障体系逐步完善，城乡最低生活保障全面落实，对外来工实行大病医疗保险。努力解决特困群众住房、就医、打官司、子女入学“四难”问题，对口扶贫工作取得实效。保护劳动者权益，依法清理欠薪。从２００１年起，市政府每年办理“十件实事”，一大批民心工程惠及广大群众。“两大整治”取得阶段性成果，市场经济秩序良好，社会治安秩序保持稳定。坚持每年建成一项大型公共文化基础设施，广泛开展群众性文化、体育活动。医疗卫生事业在改革中发展，人口和计划生育工作走在全省前列。双拥共建成绩突出，第五次蝉联“全国双拥模范城”称号。新闻出版、广播电视、外事、侨务、民族、宗教、审计、人防、档案、仲裁、气象、民兵、老年人和港澳流动渔民等工作都取得新成绩，工会、共青团、妇联、残联、科协、红十字会、工商联、文联、侨联、社科联等社会团体卓有成效的工作，促进了经济社会协调发展。

　　政府自身建设取得良好成绩。五年来，我们着眼于优化发展环境，不断加强政府自身建设。坚持依法行政，加强政府法制建设，起草一批质量较高的地方性法规草案，颁布执行一批政府规章。深入开展普法教育，强化执法监督，规范了行政行为。机构改革顺利进行，政府机构和人员大幅精简。加快转变职能，行政审批项目减少８８．２％。坚持向市人大及其常委会报告工作、向市政协通报情况，认真听取民主党派、工商联、无党派人士的意见。健全议事规则和办事程序，发挥专家咨询机构的决策参谋作用，决策科学化、民主化水平有所提高。加强公务员培训，实行公开选拔、竞争上岗制度，一批优秀年轻干部走上领导岗位。加强廉政建设，推行政务公开，开展行风评议，严厉惩治腐败。连续五年深入开展加强机关作风建设活动，规范窗口服务，加强督查督办工作，为重点项目提供现场服务，开展“万人评政府”活动，政府工作效率和服务水平不断提高。

　　各位代表，２００３年是很不平凡的一年。我们取得了防治“非典”疫情和经济社会发展的双胜利，ＧＤＰ比上年增长１８．１％，是１９９４年以来的最好成绩。“工业西进、城市西拓”初见成效，全市工业经济强劲增长。成功举办“世界经济发展宣言”系列活动，招商引资形势喜人，外贸出口增速进入全省前列。港珠澳大桥等一批关系珠海未来发展的重大交通基础设施项目取得进展，江珠高速公路、粤西沿海高速公路珠海段动工兴建，珠澳跨境工业区奠基。改革城市规划和土地资源管理体制，设立城市规划委员会和土地管理领导小组，建立土地储备制度，成功进行土地使用权公开拍卖。为民办实事取得实效，降低了车辆路桥隧道收费标准，农村税费改革全面铺开，新型农村合作医疗顺利推进，唐家湾镇、三灶镇成为首批“省级卫生镇”，年初市政府承诺办理的“十件实事”得到落实。

　　各位代表，五年来取得的成绩，是全市人民在中央、省和市委的正确领导下，团结奋斗、扎实工作的结果；离不开中央、省驻珠单位和驻珠军警部队的大力支持；也与历届市委、市政府打下的良好基础分不开。在此，我代表市人民政府，向为珠海建设付出辛劳的全市人民表示崇高的敬意！向所有关心支持珠海发展的港澳台同胞、海外侨胞和海内外各界朋友表示衷心的感谢！

　　总结过去五年的政府工作，有六条重要的实践经验。一是坚持以经济建设为中心，把发展作为第一要务，用发展的办法解决前进中的问题；二是坚持解放思想，发扬特区敢闯敢试的精神，以创新推动发展；三是因地制宜，实事求是，不断探索和完善符合珠海实际的发展路子；四是充分调动各方面的积极性，形成加快发展的合力；五是把为人民群众谋利益作为政府工作的出发点、立足点，作为加快发展的根本目的，真心实意为群众办实事；六是坚持走可持续发展之路，致力于保护环境，不断优化环境。

　　在肯定成绩的同时，必须清醒地认识到，珠海经济社会发展中还存在一些突出问题。主要是：经济实力有待增强，经济结构有待优化，经济质量和效益需要进一步提升；经济发展不平衡，区镇村经济仍相当薄弱；农民增收困难，征地农民转移就业渠道有待拓宽；交通基础设施不完善、不配套的格局尚未根本改变；城市规划和土地资源管理工作仍需加强；社会管理存在一些薄弱环节；公共卫生体系需尽快完善；部分公务员精神状态和工作作风不适应发展的需要，素质有待提高。我们要高度重视存在的问题，并采取有力措施加以解决。

　　二、今后五年的展望和工作目标

　　今后五年，是珠海抢抓历史性机遇，实现更大更快更好发展的五年。一方面，新一届政府面临着更加激烈的地区竞争带来的严峻挑战，面临着以更快的发展追赶“标兵”、以更全面更协调的发展落实科学发展观、在发展中逐步解决自身矛盾和问题的多重压力。另一方面，经济全球化和世界范围产业结构加快调整转移，“大珠三角”、“泛珠三角”加强区域合作，港珠澳大桥及铁路、港口、高速公路等大型交通基础设施动工建设，都给珠海带来重大的发展机遇，加上珠海已经具备的发展基础和优势，今后的五年将是珠海可以大有作为的重要战略机遇期。我们要进一步增强责任感、使命感和紧迫感，牢牢抓住历史性的重大机遇，同时要保持清醒的头脑，做好应对各种困难和挑战的充分准备，团结带领全市人民，努力推动珠海经济和社会发展实现新的跨越！

　　今后五年市政府工作的总体思路是：以邓小平理论和“三个代表”重要思想为指导，认真贯彻党的十六大、十六届三中全会和胡锦涛总书记视察广东重要讲话精神，按照省第九次党代会、省委九届四次全会、市第五次党代会的部署，坚持以人为本，树立科学发展观，落实“五个统筹”的要求，紧紧抓住发展第一要务，能快则快，好中求快，全力推进工业化、城市化、信息化，突出抓好产业发展、交通基础设施建设、新城区开发、珠港澳合作、农业农村和农民工作，正确处理改革发展稳定的关系，推动经济社会全面、协调、可持续发展，实现物质文明、政治文明和精神文明共同进步，为实现建设宽裕小康社会的目标和率先基本实现社会主义现代化奠定坚实的基础。

　　今后五年市政府工作的主要任务是：努力实现珠海市第五次党代会确定的“聚集高素质人才、发展高质量经济、建设高品位城市，力争在综合经济实力、产业规模和城市架构上‘再造一个新珠海’，社会发展和城市管理水平跃上新台阶”的工作目标，同时不断加强自身建设，初步建成高效清廉的服务型政府。

　　今后五年市政府工作的主要预期目标是：

　　──经济跨越式发展。在优化结构、提高质量和效益的基础上，到２００８年，全市ＧＤＰ和财政总收入分别比２００３年翻一番。

　　──城市建设迈出更大步伐。按照构筑东西“双城”框架和“提升东城、打造西城”的要求，推动西部新城组团、唐家湾－淇澳科教新城和南湾－横琴新城规划建设取得显著进展，香洲城区建设水平进一步提升，城市特色更加突出，城市辐射功能增强。

　　──人民生活整体达到宽裕小康。到２００８年，人均ＧＤＰ达到７０００美元。农民收入显著增加，社会就业比较充分，社会保障体系更加健全，社会秩序优良，人民群众安居乐业。

　　──社会事业全面协调发展。建设“文化盛市”取得显著成效，教育事业得到长足发展，建成省级“教育强市”，形成比较完善的科技创新体系和全民健身、医疗卫生体系，建立人口与计划生育工作新机制，人民的思想道德素质、科学文化素质和健康素质提高到新水平。

　　──生态环境保持优良。牢固树立绿色ＧＤＰ理念，进一步增强可持续发展能力，生态城市的环境品牌形象得到提升，成为休闲度假特色突出的“东南沿海重要的风景旅游城市”。

　　三、２００４年的工作安排

　　２００４年是新一届政府任期的第一年，我们要树立正确的政绩观，落实科学的发展观，大兴求真务实之风，认真贯彻中央确定的“稳定政策、适度调整，深化改革、扩大开放，把握全局、解决矛盾，统筹兼顾、协调发展”的工作思路，充分调动一切有利因素，聚精会神搞建设，一心一意谋发展，确保实现各项工作的良好开局。

　　今年我市经济社会发展的主要预期目标是：ＧＤＰ增长１３％；工业增加值增长１５％；财政一般预算收入增长１２．５％；社会消费品零售总额增长１１％；固定资产投资增长１１％；外贸出口增长１０％；实际利用外商直接投资增长８％常住人口自然增长率控制在７．１‰以下；城镇登记失业率控制在３％左右；城镇居民年人均可支配收入增长７％；农渔民年人均纯收入增长１０％。

　　为实现上述目标，要做好以下工作：

　　（一）狠抓工业发展，调整优化结构，增强产业实力和竞争力。

　　坚持大办工业不放松。要坚定不移地走新型工业化道路，把发展工业作为当前经济工作的重中之重来抓，进一步强化宏观引导，优化微观服务，做到领导不分心、部门齐支持、基层不松劲。

　　提高园区综合开发水平。在全市范围对工业园区进行合理布局，集中资源办好现有园区。积极创新投融资体制，多渠道吸引外资、金融资本、民间资本参与园区开发建设，加快完善基础配套设施。调整绩效考核机制，引导园区开发更加注重投资强度、更加注重产业特色、更加注重经济社会效益、更加注重环境保护。从加强规划和土地管理等关键环节着手，引导园区合理安排开发时序，做到“统一规划、滚动发展、集约建设”。鼓励园区之间错位发展，形成各有特色、优势互补、互动发展的格局，避免无序竞争。加快为工业园区配套的第三产业发展，具体思路是市管规划，区抓协调，镇为责任主体，用市场机制选择投资者，利益留在镇村。

　　推动产业结构调整升级。深入研究当前产业结构调整和新一轮产业转移的新特点、新趋势，加紧做好我市的产业发展规划。总的方向是以发展先进制造业为主导，实现轻重工业并举发展。要加快电子及通讯设备制造业、电气机械及器材制造业、医药及医疗器械制造业等现有优势产业的发展，同时抢抓我省产业结构向重型化方向调整升级的新机遇，充分发挥珠海发展重化工业的比较优势，以临港工业区等为重点区域，在发展石化、能源、装备工业等方面努力实现更大突破。

　　大力发展高新技术产业。进一步理顺珠海高新区管理体制，以科技创新海岸为重点，优化创业服务环境，加快高新区“二次创业”步伐。继续支持国家软件产业基地、清华科技园、哈工大新经济资源开发港等一批科技孵化器和科技成果产业化基地的建设，巩固和壮大电子信息产业，大力培育发展生物技术、新材料、新能源等新兴产业。特别是要争取在软件产业和集成电路制造业等方面有更大的发展。积极应用高新技术和先进适用技术改造传统产业。

　　优化企业结构。要发挥大企业、大项目对产业发展的拉动作用，高度重视引进和培育市场竞争力强、创新能力强的龙头企业。依托龙头企业，定向组织上下游产业配套招商，延长产业链条。大力扶持中小企业发展，鼓励中小企业与大型龙头企业协作配套，引导中小企业向“专、精、特、新”方向发展。增加财政贴息资金，加大对企业技术改造和技术创新的支持力度，增强企业发展后劲。实施名牌带动战略，强化产品技术标准和质量监督，依法保护企业商标权，鼓励企业树品牌、创名牌。

　　加快发展现代服务业。旅游业是我市服务业的龙头，要突出抓好旅游业发展。加快珠海温泉等大型旅游项目开发建设，积极发展海岛等特色旅游，加强与港澳及周边地区的旅游合作，优化旅游综合环境，努力建设“中国最佳旅游城市”。深入开展物流业发展规划研究，着力吸引大型物流企业落户，推进现代物流业发展。培育会展业，引进会展龙头企业，尽快建设适应发展需要的会展场馆，办好第五届中国航展、世界汽车摩托车比赛等特色展会。发展金融业，积极吸引金融企业来我市设立总部或管理机构。支持商贸业发展，加快莲花路改造，建设商业特色街区，加快西部地区大型商业设施规划建设，营造良好的商业氛围。大力发展会计审计、法律服务、信息咨询等中介服务业。

　　（二）以促进农渔民增收为中心，大力发展农业和农村经济。

　　推动农村工业化、城镇化和农业产业化。解决农业、农村和农民“三农”问题的根本出路，在于系统推进农村工业化、城镇化和农业产业化。“三化”的核心是工业化。城镇化要依托工业化，并在体制创新方面有所突破，重点是进行镇村重组，稳步推进户籍制度改革，引入规划和市政概念，提高村镇建设水平，引导农民向城镇居民转变。农业产业化的重点是引进和培育农业龙头企业，推广农业科技和现代流通方式，逐步实现基地化、订单化、标准化生产。

　　大力调整农业生产结构。大力发展水产养殖、蔬菜、花卉园林种植等高效特色农业，加快发展出口创汇农业。要充分调动农民调整生产结构的积极性，政府重点抓好基地建设、扶持龙头、农业示范、种苗繁育、技术推广、搞活流通等环节。

　　多渠道增加农民收入。今年要以转移１万名农村劳动力为目标，大力开展农村劳动力“订单式”职业技能培训，引导农民转移到第二、三产业就业，带动农民增收。大力发展农村集体经济，鼓励发展入股经营、物业出租等低风险经济，增加集体分红收入。积极探索用征地补偿金为被征地农民办理社会保障、变一次性补偿为分年度补偿等新办法，保障被征地农民的长远利益。巩固农村税费改革成果，减轻农民负担。

　　改善农村生产生活条件。加快农村公路网和公交网络建设，加大对农田基本建设和堤围等水利设施的投入，抓紧建设动植物防疫监督体系，逐步改善农村金融服务。对金湾区自来水系统进行改造，逐步解决香洲区边远村和横琴、桂山等海岛群众饮水难问题。继续改造农村电网。加快平沙、红旗危房改造进度。

　　大力发展海洋经济。加快万山海洋开发试验区综合开发步伐，增加海岛基础设施建设投入，吸引有实力的投资者开发海洋渔业、海岛旅游、中转仓储物流和海洋生物医药等产业，增加渔民和海岛居民收入，逐步改变海岛面貌。

　　（三）推动交通项目建设，努力完善城市基础设施。

　　全力推动珠海从交通末梢向交通枢纽转变。以加快构筑立体交通网络为目标，力促一批重大交通设施项目动工建设。港珠澳大桥对珠海发展关系极为重大，我们要配合各方面做好前期工作，推动尽快落实方案和动工建设。要积极争取有关方面支持，力促广珠西线高速公路尽快动工，推动广珠铁路建设，配合做好广珠城际快速轨道项目建设的前期工作。要继续做好在建的江珠高速公路、粤西沿海高速公路珠海段的服务和相关配套工作。加紧对市内多条国道、省道进行改造。加快市区与西部地区第二条通道的规划建设。

　　加快港口建设。珠海要坚定不移地走“以港兴城”的发展路子。进一步加强对港口发展的领导，理顺港口规划、发展和管理体制，强化市港口管理部门职能。落实港口发展规划，妥善处理临港工业与港口发展的关系，严格按规划管理、使用和保护岸线。要重点推动高栏港区两个五万吨级集装箱码头开工建设，全面启动集装箱码头作业区建设，加快大吨位石化公用码头建设和扩建，加快建设洪湾港。

　　逐步完善城市基础配套设施。支持供电部门新建和扩建一批变电站，改造电网设施，做好电力调度工作，千方百计保障电力供应。加快完善供水系统，确保珠澳两地正常供水。实施城乡水利防灾减灾工程，在汛期前完成草塘湾海堤等应急度汛工程，增强防洪能力。动工建设唐家、南湾污水处理厂，加快规划建设金湾区、斗门区和临港工业区污水处理设施，继续抓好城市污水管网和垃圾填埋场建设。稳步推进城市管道燃气项目建设，推广使用管道燃气。有重点地逐步完善城市消防设施。

　　（四）推进新城区开发，提高城市规划、建设和管理水平。

　　稳步推进新城区开发。加快新城区开发建设，是城市化与工业化协调发展的迫切要求。今年的工作重点一是完善规划，二是加快基础设施配套建设。

　　强化规划的龙头地位。市区两级政府都要增加规划经费投入，市级投入今年不低于１０００万元。以国务院批准的城市总体规划为指导，按照市第五次党代会的精神，加紧组织编制分区规划、控制性详细规划和市政规划，特别是要抓紧完善和深化唐家湾、南湾、横琴和西部新城组团的发展规划。规划编制要坚持高标准，建立健全重大规划项目的公众参与、专家评审、政府决策、人大政协审议监督的“四位一体”机制。要加强规划管理，依法维护规划的权威性、严肃性。

　　坚持基础设施先行。拓展新城区，要以公共基础设施先行建设为重点，构筑良好的开发平台，形成投资的追随效应。今年要加快建设情侣北路和南横路，带动唐家湾－淇澳科教新城和南湾－横琴新城的发展。依托西部地区工业的发展，加快规划建设磨刀门以西的井岸－白蕉新城、西湖－三灶新城、港区新城、珠港新城等新城市组团。

　　加强土地资源管理。要十分珍惜宝贵的土地资源，加大力度清理并妥善处理闲置土地，进行土地盘整，发挥土地储备制度的作用，提高政府对土地资源的调控能力。合理进行房地产市场宏观调控，引导有实力的房地产商参与新城区开发建设，促进房地产业持续健康发展。

　　提高城市经营管理水平。加快香洲区城中旧村和老工业区改造，积极支持盘活烂尾楼。实施“生态家园”十年植树规划，建设林荫大道，提高绿化水平。整治市容市貌，高水平改造海滨泳场，加快前山河整治，建设灯光工程，营造新的城市景观。依法查处违法违规建设行为，逐步清拆违章建筑。落实《物业管理条例》，提高物业管理水平。进一步理顺城市管理体制，继续推进相对集中行政处罚权工作，加强城市管理法规建设，规范执法行为。对口岸地区、旅游景点等重点区域进行综合整治，提高管理效能。积极探索开展城市公共资源经营。

　　（五）深化改革，逐步完善社会主义市场经济体制。

　　坚持社会主义市场经济的改革方向。认真贯彻《中共中央关于完善社会主义市场经济体制若干问题的决定》，继续深化体制改革，为经济和社会发展提供强有力的体制保障。

　　深化国有经济改革。完善管人、管事、管资产相结合的国有资产管理体制，实施国有企业战略性重组，推动国有资本向先导性、基础性、公益性领域和优势产业集中。加快产权制度改革，使股份制成为公有制的主要实现形式，逐步建立归属清晰、权责明确、保护严格、流转畅顺的现代产权制度，发展混合所有制经济。在具有行业垄断特点的国有企业率先进行产权多元化试点。清理非经营性国有资产，逐步实现集中统一管理，提高使用和运营效益。

　　大力发展民营经济。认真落实省、市扶持民营经济发展的政策措施，支持和鼓励民营资本进入法律法规未禁止的一切行业和领域，凡是对外资开放的，也对民营资本开放。坚持一手抓扶持服务，一手抓规范提高，促进生产型、科技型、外向型、吸纳就业型、农产品加工型民营企业加快发展。

　　推进财税、金融和投融资体制改革。深化以国库集中支付为核心的财政管理体制改革，坚持量入为出、科学理财，优化财政支出结构，保持支出水平与经济发展水平相适应，不用“过头钱”。规范非税收入管理，全面实行部门综合预算，强化预算约束。依法治税，做到应收尽收，但不收“过头税”。贯彻《国务院关于积极推进资本市场改革开放和稳定发展的意见》，积极培育上市公司，扩大资本市场融资规模。设立地方金融服务机构，继续防范和化解金融风险。全面推行社会投资基本建设项目登记备案制度，确立企业的投资主体地位，扩大社会投资规模。严格执行《珠海市政府投资项目管理条例》，进一步规范政府投资行为，加强项目绩效审计，提高投资效益。

　　继续整顿和规范市场经济秩序。组建有形要素市场管理机构，提高产权交易中心等七个中心的规范运作水平。开展各类专项整治，全面实施食品和药品放心工程，严厉打击走私贩私、制假售假、偷逃骗税、非法传销、商业欺诈等违法犯罪行为。规范网吧经营。继续清理债权、债务，妥善处理历史遗留问题。建设“诚信政府”，加快社会信用体系建设。

　　（六）扩大开放，推动区域经济合作。

　　大力招商引资。加强统筹指导，整合招商资源，培育专业化、市场化的招商队伍。创新招商方式，办好海岛招商、服务业招商等专题招商活动。在继续吸引外资投资制造业的同时，扩大现代服务业利用外资规模。在继续吸引港澳台投资的同时，加大对日韩欧美等国家和地区的招商力度。进一步强化对内招商，大力吸引内地大中型国有企业、上市公司和民营企业的投资。积极吸引企业技术研究开发机构和管理总部落户珠海。

　　拓展国内外市场。适应出口退税政策调整，推进外贸主体多元化，努力扩大出口。坚持科技兴贸，以质取胜，优化出口商品结构。支持扩大进料加工产品出口，引导加工贸易转型升级，提高增值率。努力扩大服务贸易出口。支持优势企业“走出去”对外投资、承包工程和开展劳务合作。加强与内地大中型城市多种形式的合作，鼓励珠海企业参与西部大开发和东北老工业基地振兴，实现优势互补，拓展内地市场。着力保护消费者权益，优化消费环境，增加居民即期消费。

　　推进“大通关”建设。抓好口岸设施建设和改造，重点改善横琴口岸设施条件。加强与中央和省驻珠海口岸查验单位的沟通，推动口岸管理改革，逐步采用现代化查验方式，提高通关效率。

　　大力推进珠港澳合作。抢抓内地与香港、澳门关于建立更紧密经贸关系的安排（ＣＥＰＡ）实施的先机，全面推进珠港澳新一轮合作。在中央和省的指导下，尽快落实货物贸易、服务贸易和投资贸易便利化方面更紧密合作的措施，吸引港澳服务业进入珠海，支持建设ＣＥＰＡ概念的商业街区。加快珠澳跨境工业区的开发建设和珠澳合作开发横琴岛的步伐。积极支持珠海机场对外合作。加强与台湾经济技术交流与合作。

　　（七）发展科技教育事业，增强城市发展后劲。

　　优化科技创新环境。加快建立政府推动与企业为主体相结合、产学研相结合、引进与自主创新相结合的技术创新体系。政府重点是强化公共服务平台建设，逐步完善科技支撑体系和服务体系，努力营造适合科技创业的环境和氛围。继续加大对科技事业的投入，加强科技经费管理，提高使用效益。

　　加快发展基础教育。要把教育放在优先发展的地位。加大市、区两级财政对基础教育的投入，全面启动“教育强市”工程，高质量、高水平普及九年义务教育。着力解决西部地区和城区学位不足问题，逐步解决流动人口子女入学问题。加强教师队伍建设，推进省级示范性小学、示范性初中和国家级示范性普通高中创建工作，继续增加中小学优质教育学位。重视农村教育和幼儿教育。大力发展职业教育和继续教育，整合资源，加快市城市职业技术学院建设。进一步规范和发展民办教育。积极稳妥地推进学校后勤服务社会化改革。

　　努力办好大学园区。逐步提升已引进高校的办学层次和办学水平，促进高等教育和科技创新紧密结合，引导大学的学科设置与我市以及珠三角重点发展的产业相结合。发挥大学园区的综合效应，为珠海经济和社会发展提供人才和智力支持，使高校引得进，办得好，用得上。

　　提高信息化水平。按照“统一规划、分步实施，资源共享、重在应用”的思路，加快实施电子政务工程，以政府信息化带动企业和社会信息化，提高国民经济和社会信息化水平。

　　做好人才工作。贯彻全国人才工作会议精神，牢固树立人才是第一资源的观念，加强人才队伍建设，合理配置人才资源。实施人才居住证和政府雇员制度，吸引各类人才来珠海，努力为人才施展才干创造良好的环境。

　　（八）加强环境保护，促进人与自然和谐发展。

　　发展绝不以牺牲环境为代价。经济发展特别是工业的快速发展，客观上对珠海的生态环境构成压力。我们对此要有清醒的认识，进一步增强环境意识，更加注重环境保护。

　　加强生态环境保护。落实环保责任制，市对区、经济功能区的考核实行经济指标与环保指标并重。编制生态城市发展规划，科学制订生态环境功能区划。抓好工业园区区域环境评价，严格按照产业规划把好引进项目的环保关，重大项目环境评价情况对公众公开，加强工业污染防治工作。开展城市环境综合整治，着重治理乱排放生活污水、机动车尾气和噪声污染。加大环境监管和执法力度，实行省控重点污染源在线实时监测。继续进行珠江口综合整治，加强水环境保护。保护湿地，建设好淇澳红树林保护区。进一步保护海洋生态环境。

　　增强可持续发展能力。加强宣传教育，增强全社会环境保护意识。推广清洁生产、资源综合利用技术，发展绿色经济、循环经济，逐步建设节约型社会。积极发展环保产业。

　　加强人口与计划生育工作。贯彻国家和省人口与计划生育政策法规，认真落实目标管理责任制，强化优质服务，稳定低生育水平，提高出生人口素质。加强流动人口计划生育综合治理。

　　（九）推进“文化盛市”建设，发展社会各项事业。

　　提高公民思想道德素质。深入开展群众性精神文明创建活动，大力弘扬“爱国守法、明礼诚信、团结友善、勤俭自强、敬业奉献”的基本道德规范。以青少年为重点，加强全社会科普教育，引导居民养成科学、健康、文明的生活方式。

　　加快文化基础设施建设。重点支持公益性文化事业和文化基础设施建设。完成市图书馆和市青少年活动中心、市妇女儿童活动中心工程建设，尽快为市民提供优良服务。尽快动工建设珠海美术馆（古元艺术馆），推动中央音乐学院珠海校区音乐厅加快建设。加快镇特级文化站建设。进一步发掘历史文化资源，做好文物古迹保护和开发利用工作。

　　发展文化产业和繁荣文化事业。实施《珠海市建设文化盛市规划纲要》，制定文化产业发展规划，着力培育和扶持文化龙头企业和龙头产业，落实相关文化经济政策，引导文化与经济融合。推进文化事业单位改革，积极探索建立适应先进文化发展规律和市场要求的新体制。整合广播电视资源，推动广播电视体制改革。鼓励文艺创作，多出文艺精品。

　　推动各项社会事业全面协调发展。实施“小康体育工程”，广泛开展全民健身活动，积极承办重要的国际体育赛事。大力发展体育产业。认真实施妇女儿童发展纲要，加快妇女儿童事业发展。做好国防动员、民兵预备役、双拥共建和优抚安置工作。继续做好民族、宗教、人防、国安、对台、外事、侨务、统计、档案、气象、工会、老年人、残疾人和港澳流动渔民等工作。

　　（十）坚持以人为本，切实改善人民群众生活。

　　加强就业和再就业工作。就业是民生之本，要把实现比较充分的就业作为政府优先工作目标。以增加就业岗位为目标，以落实再就业政策为主线，以强化再就业服务为手段，以加大资金投入为保障，完善就业服务体系，加强就业技能培训、职业介绍和就业指导。努力改善创业和就业环境，鼓励、支持自主创业和灵活就业。做好困难职工就业援助和帮扶工作。

　　进一步完善社会保障体系。扩大社会保险覆盖面，重点抓好非公有制企业员工和灵活就业人员的参保工作。探索建立原村民转居民和征地农民养老保险制度。做好企业退休人员进行社区化管理工作。加强社保基金征缴和监管，提高基金营运水平。加大劳动保障监察执法力度，保障全体劳动者的合法权益。

　　提高公共卫生保障水平。继续做好“非典”、高致病性禽流感等疫病的防控工作。筹建市传染病医院，加紧建立健全突发公共卫生事件应急机制、疾病预防控制体系、卫生监督执法体系和医疗救治体系。加快市人民医院、市妇幼保健院和拱北医院改扩建工程。重视做好农村公共卫生、医疗服务体系建设和医药卫生监督工作，大力开展创建卫生村、镇活动。加强医德医风建设，提高医疗服务水平。

　　强化安全生产监管。认真落实《安全生产法》，加强全民安全教育，深入开展安全生产大检查和专项整治，努力预防和减少安全事故发生，确保人民群众的生命财产安全。

　　关心群众生产生活。认真实施“十项民心工程”，积极帮助城乡困难群众解决基本生活问题。落实最低生活保障制度，对符合条件的城乡困难居民做到“应保尽保”。做好法律援助工作，维护群众的合法权益。加强城市生活无着流浪乞讨人员救助管理。

　　（十一）坚持依法治市，建设为民、务实、清廉政府。

　　加强法制建设。认真贯彻实施《行政许可法》，规范行政许可行为，进一步推进依法行政。加快起草一批重要的地方性法规草案和政府规章，提高政府立法工作质量。落实行政执法责任制和过错追究制度，严格执法监督。加强司法、仲裁和行政复议工作。

　　维护社会稳定。坚持标本兼治，综合治理，开展打击抢劫、抢夺和入室盗窃等专项整治行动，大力扫除“黄、赌、毒”等社会丑恶现象，建立和完善打、防、控一体化的社会治安长效机制，增强人民群众的安全感。做好群众来信来访工作。

　　转变政府职能。进行新一轮政府机构改革，合理界定政府职能，公共资源更多地向社会管理和公共服务倾斜。政府不该管、管不好的事要交给企业、社会组织和中介机构。同时加强对中介机构的规范和监管。政府该管的事一定要管好，并要不断改进管理方式和方法，提高行政效能和工作效率。深化事业单位改革。合理调整市与区的职能关系，落实简政放权等措施，充分调动区政府和镇、街道办的积极性。

　　科学民主决策。坚持定期向市人大及其常委会报告工作，自觉接受市人大及其常委会的监督。积极支持人民政协履行政治协商、民主监督、参政议政职能，主动听取各民主党派、工商联、无党派人士的意见和建议。认真办好“两案一议”。主动接受人民群众和新闻舆论对政府工作的监督，充分发挥工会、共青团、妇联等人民团体的桥梁和纽带作用。遵循科学规律，建立和完善重大问题集体决策制度、专家咨询制度、社会公示和听证制度，提高决策的透明度和公众参与度。继续全面推行政务公开制度。

　　进一步改进作风。坚持“两个务必”，弘扬求真务实精神，不搞“政绩工程”、“形象工程”，不图虚名，埋头实干、少说多干。大力精简会议和文电。继续加强督查督办工作，推动各项决策落到实处。抓好政风建设，坚决纠正损害群众利益的不正之风。加强公务员廉洁从政、干净干事的教育，加大廉政监察和行政效能监察力度，从源头上预防和治理腐败，坚决惩处腐败分子，努力建设一支“为民、务实、清廉”的公务员队伍。

　　（十二）继续办好服务于广大人民群众的十件实事。

　　一是启动相关工程建设，进一步完善全市供水系统。

　　二是实现行政村全部通水泥路。

　　三是实施农村富余劳动力转移就业工程。

　　四是提高新型农村合作医疗的覆盖率和保障水平。

　　五是建设疫情和突发公共卫生事件应急信息系统。

　　六是完成三台石路、柠溪西路续建工程。

　　七是建设凤凰山广播电视发射台。

　　八是扩建市第三中学。

　　九是改造南屏至横琴大桥的南横路。

　　十是改善临港工业区和前山地区消防保障条件。

　　各位代表！今后五年和今年的政府工作任务艰巨而光荣。党的十六大和十六届三中全会为改革开放和现代化建设指明了方向，省委、省政府和市委对珠海发展作出了全面部署。市政府将团结、带领和依靠全市人民，高举邓小平理论和“三个代表”重要思想伟大旗帜，紧密地团结在以胡锦涛同志为总书记的党中央周围，解放思想，实事求是，与时俱进，开拓创新，扎实工作，为取得珠海改革开放和现代化建设的新胜利而努力奋斗！
